# Interplay between lncRNA *RP11-367G18.1* variant 2 and YY1 plays a vital role in hypoxia-mediated gene expression and tumorigenesis

**DOI:** 10.1186/s12935-023-03067-6

**Published:** 2023-11-08

**Authors:** Pei-Hua Peng, Ji-Lin Chen, Heng-Hsiung Wu, Wen-Hao Yang, Li-Jie Lin, Joseph Chieh-Yu Lai, Jeng-Shou Chang, Jia-Ling Syu, Han-Tsang Wu, Fei-Ting Hsu, Wei-Chung Cheng, Kai-Wen Hsu

**Affiliations:** 1 Cancer Genome Research Center, Chang Gung Memorial Hospital at Linkou, Taoyuan, 333 Taiwan; 2 Comprehensive Breast Health Center, Taipei Veterans General Hospital, No. 201, Sec. 2, Shih- Pai Road, Taipei, 112 Taiwan; 3 Research Center for Cancer Biology, China Medical University, Taichung, 40402 Taiwan; 4 Drug Development Center, Program for Cancer Biology and Drug Discovery, China Medical University, Taichung, 40402 Taiwan; 5 Graduate Institute of Biomedical Sciences, China Medical University, Taichung, 40402 Taiwan; 6 The PhD Program for Cancer Biology and Drug Discovery, China Medical University and Academia Sinica, Taichung, 40402 Taiwan; 7 Institute of Biomedical Science, China Medical University, Taichung, 40402 Taiwan; 8 Cancer Research Center, Changhua Christian Hospital, Changhua, 500 Taiwan; 9 Department of Biological Science and Technology, China Medical University, Taichung, 40402 Taiwan; 10 Institute of Translational Medicine and New Drug Development, China Medical University, Taichung, 40402 Taiwan

**Keywords:** Hypoxia, lncRNA *RP11-367G18.1* variant 2, YY1

## Abstract

**Background:**

The hypoxia-responsive long non-coding RNA, *RP11-367G18.1*, has recently been reported to induce histone 4 lysine 16 acetylation (H4K16Ac) through its variant 2; however, the underlying molecular mechanism remains poorly understood.

**Methods:**

RNA pull-down assay and liquid chromatography-tandem mass spectrometry were performed to identify *RP11-367G18.1* variant 2-binding partner. The molecular events were examined utilizing western blot analysis, real-time PCR, luciferase reporter assay, chromatin immunoprecipitation, and chromatin isolation by RNA purification assays. The migration, invasion, soft agar colony formation, and in vivo xenograft experiments were conducted to evaluate the impact of *RP11-367G18.1* variant 2–YY1 complex on tumor progression.

**Results:**

In this study, RNA sequencing data revealed that hypoxia and *RP11-367G18.1* variant 2 co-regulated genes were enriched in tumor-related pathways. YY1 was identified as an *RP11-367G18.1* variant 2-binding partner that activates the H4K16Ac mark. YY1 was upregulated under hypoxic conditions and served as a target gene for hypoxia-inducible factor-1α. *RP11-367G18.1* variant 2 colocalized with YY1 and H4K16Ac in the nucleus under hypoxic conditions. Head and neck cancer tissues had higher levels of *RP11-367G18.1* and YY1 which were associated with poor patient outcomes. *RP11-367G18.1* variant 2–YY1 complex contributes to hypoxia-induced epithelial–mesenchymal transition, cell migration, invasion, and tumorigenicity. YY1 regulated hypoxia-induced genes dependent on *RP11-367G18.1* variant 2.

**Conclusions:**

*RP11-367G18.1* variant 2–YY1 complex mediates the tumor-promoting effects of hypoxia, suggesting that this complex can be targeted as a novel therapeutic strategy for cancer treatment.

**Supplementary Information:**

The online version contains supplementary material available at 10.1186/s12935-023-03067-6.

## Background

Long non-coding RNAs (lncRNAs), transcripts longer than 200 nucleotides with limited protein-coding potential, play a critical role in gene regulation. LncRNAs participate in multiple processes regulating gene expression, such as chromatin organization via histone modification, transcription factor recruitment, and maintenance of mRNA stability [1]. Increasing evidence has revealed the crucial roles of lncRNAs in various cellular processes, such as cell migration, stemness, and genome maintenance [2]. Dysregulation of lncRNAs can drive tumor progression and serve as a prognostic marker [3, 4].

Hypoxia, deficiency of oxygen, is a common condition in solid tumors that facilitates tumor growth, angiogenesis, and metastasis. Hypoxia-inducible factors (HIFs) are the key transcriptional regulators of gene expression. HIF-1α is prominently upregulated under hypoxia and modulates the epithelial–mesenchymal transition (EMT)-activating transcription factors, histone modifiers, and lncRNAs [5–7]. Several hypoxia-responsive lncRNAs have been reported to mediate HIF-1α signaling via diverse mechanisms [8]. These hypoxia-responsive lncRNAs interact with protein complexes, epigenetic regulators, and microRNAs to regulate hypoxic gene expression [9]. Although lncRNAs have been reported to guide chromatin-modifying complexes, lncRNA-associated histone marks are not yet fully understood.

Emerging role of the alternative splicing of lncRNAs has gained attention in cancer research. LncRNAs can undergo alternative splicing to produce different variants having different functional mechanisms and regulate tumorigenesis in a transcript-dependent manner [10]. Recently, we identified a hypoxia-responsive lncRNA, *RP11-367G18.1* (ENSG00000230943), that is associated with poor outcomes in patients with head and neck squamous cell carcinoma (HNSC) [11]. Variant 2 of *RP11-367G18.1* (ENST00000452675.1) is a key regulator of EMT and histone 4 lysine 16 acetylation (H4K16Ac). The interacting partner of *RP11-367G18.1* variant 2, which mediates its function under hypoxia, remains ambiguous. In this study, we found that the *RP11-367G18.1* variant 2–YY1 complex contributes to hypoxia-induced H4K16Ac activation and cancer progression.

## Methods

### Cell culture

Human cell lines with low (MCF7 breast cancer and FADU HNSC cell lines) and high (H1299 non-small lung cancer and MDA-MB-231 breast cancer cell lines; Figure S1A) HIF-1α levels were purchased from the American Type Culture Collection (Manassas, VA, USA). All cell lines were cultured in Dulbecco’s Modified Eagle’s Medium (Thermo Fisher Scientific, Waltham, MA, USA) supplemented with 10% fetal bovine serum at 37 °C and 5% CO_2_. For hypoxic conditions, cells were cultured in 1% O_2_, 5% CO_2_, and 94% N_2_ for 18 h. Cells were then tested for mycoplasma.

### Plasmid construction

Expression constructs for *RP11-367G18.1* variant 2 were constructed as previously described [11]. Plasmids pHA-HIF-1α, pHA-HIF-1α (ΔODD), and pHA-HIF-1α (LCLL) expressing wild-type and mutant HIF-1α were obtained from Dr. L. E. Huang (University of Utah, Salt Lake City, UT, USA) [12]. Expression construct Myc-DDK-tagged-YY1 containing cDNA encoding YY1 was cloned into a pCMV6-Entry vector. For knockdown experiments, the target sequences of *RP11-367G18.1* variant 2 (5’-GGTTCTACTTCCTGGCAAGTA-3’), *RP11-367G18.1* variant 1 (5’- GGTCCTCTTCAATGTACAATC-3’), YY1 (5’-GCCTCTCCTTTGTATATTATT-3’), HIF-2α (5’-CAGTACCCAGACGGATTTCAA-3’), and Scrambled control (5’-CCTAAGGTTAAGTCGCCCTCG-3’) were cloned into pLV2-U6-Puro and pLKO.1-puro vectors, respectively. To generate reporter constructs, the YY1 promoter fragments were cloned into a pGL3-basic vector. Hypoxia response element (HRE) mutants of the reporter constructs were cloned using QuikChange Lightning (Agilent, Santa Clara, CA, USA).

### Transfection, luciferase reporter assay, and lentivirus-mediated gene knockdown

Cells were seeded overnight and transfected using Lipofectamine 2000 (Thermo Fisher Scientific), following the manufacturer’s instructions. For the luciferase reporter assay, the reporter constructs and HIF-1α-expressing constructs were co-transfected into FADU cells under normoxia or hypoxia. Luciferase activity was measured using the Dual Luciferase Reporter Assay System (Promega, Madison, WI, USA) and further normalized to *Renilla* luciferase activity. For knockdown experiments, plasmids containing short hairpin RNA were co-transfected with pMD.G and pCMVΔR8.91 plasmids into HEK293T cells for 48 h to generate a lentivirus, as previously described [13]. To generate stable clones, cells were infected with lentivirus for 24 h and selected with puromycin for two weeks.

### RNA-sequencing (RNA-seq) and data analysis

Briefly, RNA from treated cells was extracted using RNeasy, and the TapeStation System (Agilent, Santa Clara, CA, USA) was used for RNA quality control. RNA-seq libraries were generated using a KAPA Hyperprep Kit containing RiboMinus. The libraries were then sequenced via 150 nucleotide paired-end running on Illumina HiSeq/Illumina Novaseq/MGI2000 instrument. The reads were mapped to the reference GRCh38 using HISAT2 [14]. To define differentially expressed genes, we set up a cut-off of fold-change ≥ 1.5 and a false-discovery rate < 0.05. Gene Ontology (GO) analysis, including biological process, cellular component and molecular function categories, was conducted for functional annotation. Hallmark pathway enrichment analysis was conducted via Gene Set Enrichment Analysis (GSEA).

### RNA pull-down assay

RNA pull-down assay was performed using a previously described protocol, with minor modifications [15]. Briefly, biotin-labeled *RP11-367G18.1* variant 2 was transcribed using the Biotin RNA Labeling Mix and T7 RNA polymerase. Biotinylated *RP11-367G18.1* variant 2 was treated with RNaseOUT and purified using an RNeasy Mini Kit (QIAGEN, CA, USA). Twenty µg of RNA was mixed with 50 µL of streptavidin beads in an RNA capture buffer (20 mM Tris-HCl [pH 7.5], 1 M NaCl, and 1 mM EDTA) for 30 min at room temperature. Beads were then washed with NT2 buffer, added to the cell extracts, and incubated at 4 °C. After 6 h of incubation, the mixture was washed thrice. Samples were eluted and resolved via sodium dodecyl sulfate-polyacrylamide gel electrophoresis (SDS-PAGE). *RP11-367G18.1* variant 2-specific bands were excised and subjected to mass spectrometry (MS) analysis.

### Liquid chromatography-tandem MS (LC-MS/MS) analysis and protein identification

Biotinylated sense *RP11-367G18.1* variant 2-specific bands were excised and trypsinized for peptide extraction. LC-MS experiments were performed using an LTQ-Orbitrap Fusion mass spectrometer (Thermo Fisher Scientific). The peptide mixtures were reconstituted in buffer A (0.1% formic acid) and treated on a C18 column (75 μm × 250 mm). Peptides were separated using 3 μm C18-AQ particles (100 μm × 15 cm) and mobile phase A (water with 0.1% formic acid) and a segmented gradient in 120 min up to 80% mobile phase B (acetonitrile with 0.1% formic acid) at a rate of 500 nL/min. Survey scans with 120,000 resolution and a mass range of m/z 300–1600 were performed in a data-dependent mode, and the top 10 precursors were selected. Peptide sequences were searched for trypsin specificity with 0–2 missed cleavage sites. A precursor ion mass tolerance of 10 ppm and fragment ion mass tolerance of 0.6 kDa were used. Variable modifications included carbamidomethylation and oxidation. The data were processed using MaxQuant software and filtered with a false-discovery rate of 1%.

### RNA fluorescent in situ hybridization (FISH) and immunofluorescence staining

Cells were fixed in 4% formaldehyde for 15 min and permeabilized in phosphate-buffered saline (PBS) containing 0.1% Triton X-100. Hybridization was performed using FAM dye-labeled *RP11-367G18.1* variant 2 probes (Table S1) at 37 °C overnight. For colocalization analysis, after RNA FISH, cells were fixed with 2% formaldehyde and incubated with primary antibodies against H4K16Ac or YY1 at 4 °C overnight. The next day, cells were washed thrice with PBS and incubated with the Alexa Fluor-594-labeled secondary antibody (1:2000 dilution; Abcam, Cambridge, UK) for 1 h, followed by 4′,6-diamidino-2-phenylindole staining (1:1000 dilution; Invitrogen). Cells were then observed under a Leica TCS SP8X confocal microscope.

### The Cancer Genome Atlas (TCGA) data analysis

Clinical data on gene expression in patients with HNSC were downloaded and analyzed [16, 17]. For survival analysis, the overall survival of patients was examined using the Kaplan–Meier method and compared using the log-rank test. All analyses were conducted using Prism version 8.01 (Graph Pad Software Inc., CA, USA).

### Western blot analysis and quantitative real-time PCR

Proteins and histones were extracted from the cells, and the protein concentration was measured using the Bradford method, as previously described [11]. Western blot analysis was performed using SDS-PAGE with antibodies against HIF-1α, N-cadherin (BD Biosciences, Bedford, MA, USA), E-cadherin, HA, histone H3, HIF-2α, LDHA (Cell Signaling Technology, Danvers, MA, USA), YY1 (Santa Cruz Biotechnology, TX, USA), vimentin, Flag (Sigma-Aldrich, St Louis, MO, USA), plakoglobin, H4K16Ac, Glut1 (Millipore, Burlington, MA, USA), Streptavidin (HRP), H4K5Ac, H4K8Ac, H4K12Ac, and histone H4 (Abcam), and β-actin (Genetex, Alton Pkwy Irvine, CA, USA). To determine the transcript expression levels, RNA was purified using TRIzol reagent and cDNA was synthesized using the MultiScribe Reverse Transcriptase system. Quantitative real-time PCR was performed using Fast SYBR Green Master Mix (Thermo Fisher Scientific) as previously described [18]. All primers used for quantitative real-time PCR are listed in Table S2 and S5. Relative expression levels were normalized to that of 18 S rRNA.

### Transwell migration, invasion, and soft agar colony formation assays

For the migration assay, cells (3 × 10^4^) in a serum-free medium were seeded into the upper transwell chamber with 8-µm pores and complete medium was added to the lower chamber for 12 h incubation. For the invasion assay, cells (5 × 10^4^) in a serum-free medium were seeded onto Matrigel-coated Transwell (Becton Dickinson, Mountain View, CA, USA) and incubated for 20 h. Migrated or invaded cells were fixed with methanol and stained with crystal violet (Sigma-Aldrich). For the colony formation assay, cells were plated at 5,000 cells/well on soft agar, as previously described [19]. After 14 days of incubation, cells were stained with crystal violet and counted under a light microscope.

### Chromatin immunoprecipitation (ChIP) and chromatin isolation by RNA purification (ChIRP) assays

ChIP assays were performed using anti-IgG, anti-H4K16Ac, and anti-YY1 antibodies, as previously described [11, 13]. For the ChIRP assay, anti-sense oligonucleotide probes corresponding to *RP11-367G18.1* variant 2 and LacZ were designed using a probe designer (https://www.biosearchtech.com/support/tools/design-software/stellaris-probe-designer; Table S3). Anti-sense probes were synthesized with a biotin tag at the 3′-end, as previously described [20]. Cells were harvested, rinsed with PBS, and cross-linked in 1% glutaraldehyde/PBS for 10 min at room temperature. One-tenth volume of 1.25 M glycine was added to the reaction and incubated at room temperature for 5 min. Cells were washed twice and resuspended in Pierce IP Lysis Buffer containing Protease Inhibitor Cocktail and SUPERase•In RNase Inhibitor (Thermo Fisher Scientific). Lysates were sonicated using a Bioruptor at 4 °C for 30 min and subjected to ChIRP. Probes (100 pmol) were mixed with 1 mL of the cell lysate and incubated for 4 h at 37 °C with shaking. C-1 magnetic beads (Invitrogen) were washed twice with the lysis buffer and added to the cell lysates for 60-min hybridization at 37 °C. Beads were then washed for RNA and DNA isolation. DNA samples were examined via quantitative real-time PCR using specific primers (Table S4).

### In vivo xenograft experiments

All animal experiments were approved by the Institutional Animal Care and Use Committee (2020122504) of the Chang Gung Memorial Hospital. FADU cells (2 × 10^6^) were subcutaneously injected into five-week-old BALB/c nu/nu mice (National Science Council Animal Center, Taipei, Taiwan; n = 5 per group). After inoculation for 30–35 days, the xenografted mice were sacrificed for tumor volume evaluation.

### Statistical analysis

All experiments were performed at least thrice. Error bars represent the standard deviation (SD) of the data. Statistical comparisons were made using Student’s *t*-test. *P*-value < 0.05 was considered to be statistically significant.

## Results

### Hypoxia- and *RP11-367G18.1* variant 2-upregulated genes are involved in cancer progression

**Fig. 1:**
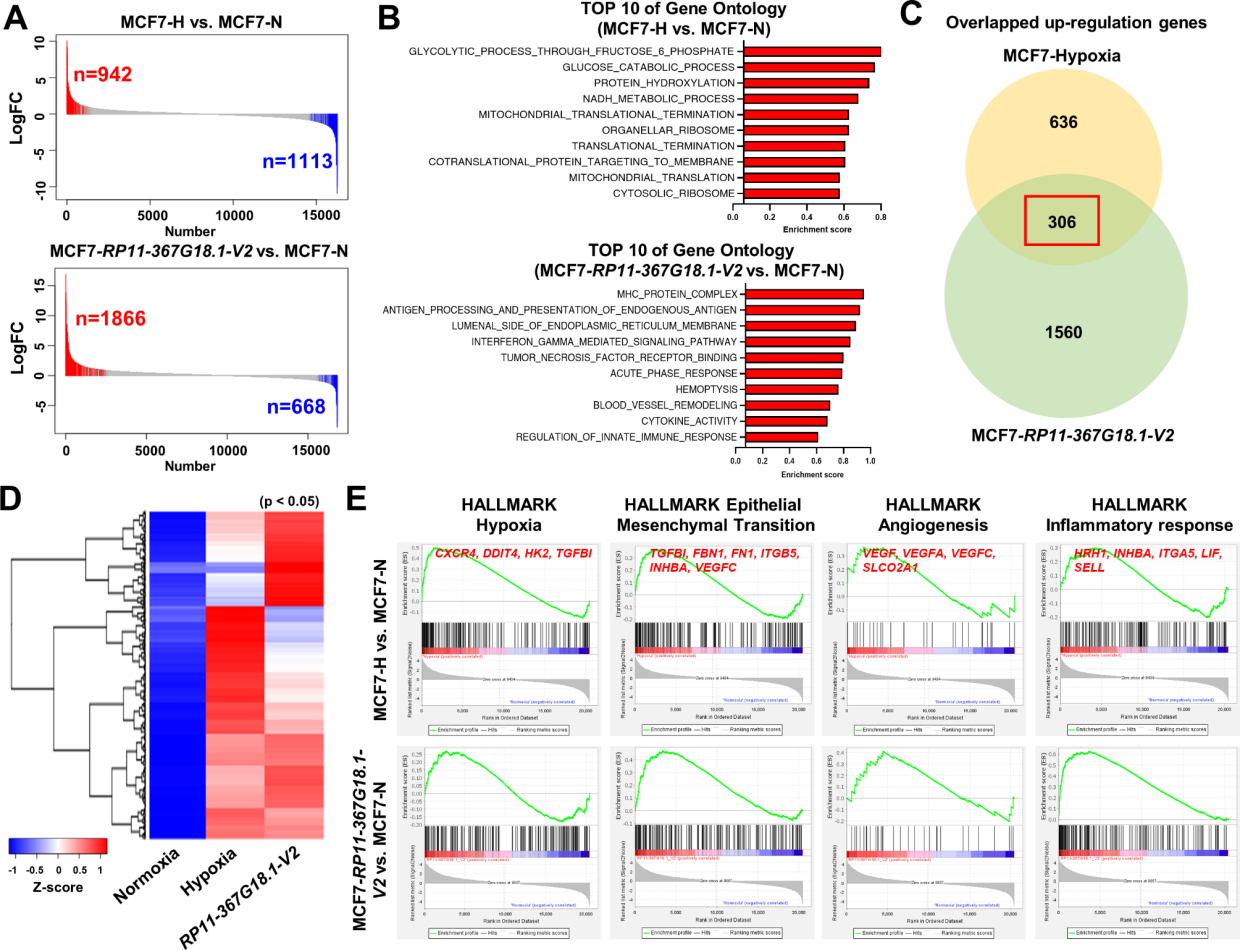
Hypoxia and *RP11-367G18.1* variant 2 co-upregulated genes involve in tumor progression. (**A**) RNA-seq revealed that genes were differentially expressed following hypoxia (up) and *RP11-367G18.1* variant 2 overexpression (bottom) in MCF7 cells. (**B**) Bar charts show the GO analysis results of upregulated genes under hypoxia (up) and *RP11-367G18.1* variant 2 overexpression (bottom). (**C**) Venn graph showed the number of hypoxia- and *RP11-367G18.1* variant 2-upregulated genes in MCF7 cells. (**D**) Heatmap analysis of hypoxia and *RP11-367G18.1* variant 2 co-upregulated genes (n = 306). (**E**) GSEA revealed that hypoxia and *RP11-367G18.1* variant 2 co-upregulated genes were enriched in the hypoxia, EMT, angiogenesis, and inflammatory response pathways. For hypoxic conditions, cells were cultured in 1% O_2_, 5% CO_2_, and 94% N_2_ for 18 h. FC, fold-change; N, normoxia; H, hypoxia; V2, variant 2

To explore the biological role of hypoxia-induced *RP11-367G18.1* variant 2, RNA-seq was performed to identify the differentially expressed genes. RNA-seq revealed that 2,055 genes (942 upregulated and 1,113 downregulated) were significantly different between hypoxic and normoxic conditions in MCF7 cells. A total of 2,534 genes (1,866 upregulated and 668 downregulated) were differentially expressed in MCF7 cells overexpressing *RP11-367G18.1* variant 2 and control cells (Fig. 1A). Upregulated genes were then classified via GO analysis. Hypoxia-upregulated genes were involved in glycolytic, glucose catabolic, and protein hydroxylation processes. *RP11-367G18.1* variant 2-upregulated genes were associated with the MHC protein complex, antigen processing and presentation of endogenous antigen, and lumenal side of endoplasmic reticulum membrane (Fig. 1B). We identified 306 genes that were upregulated by both hypoxia and *RP11-367G18.1* variant 2 overexpression (Fig. 1C and D). These upregulated genes were located in the cytoplasm, involved in the immune response, and mainly functioned in receptor binding (Figure S1B). Moreover, GSEA revealed that hypoxia- and *RP11-367G18.1* variant 2-upregulated genes were enriched in the hypoxia, EMT, angiogenesis, and inflammation pathways (Fig. 1E).

### HIF-1α-regulated YY1 interacts with *RP11-367G18.1* variant 2 to activate H4K16Ac

**Fig. 2:**
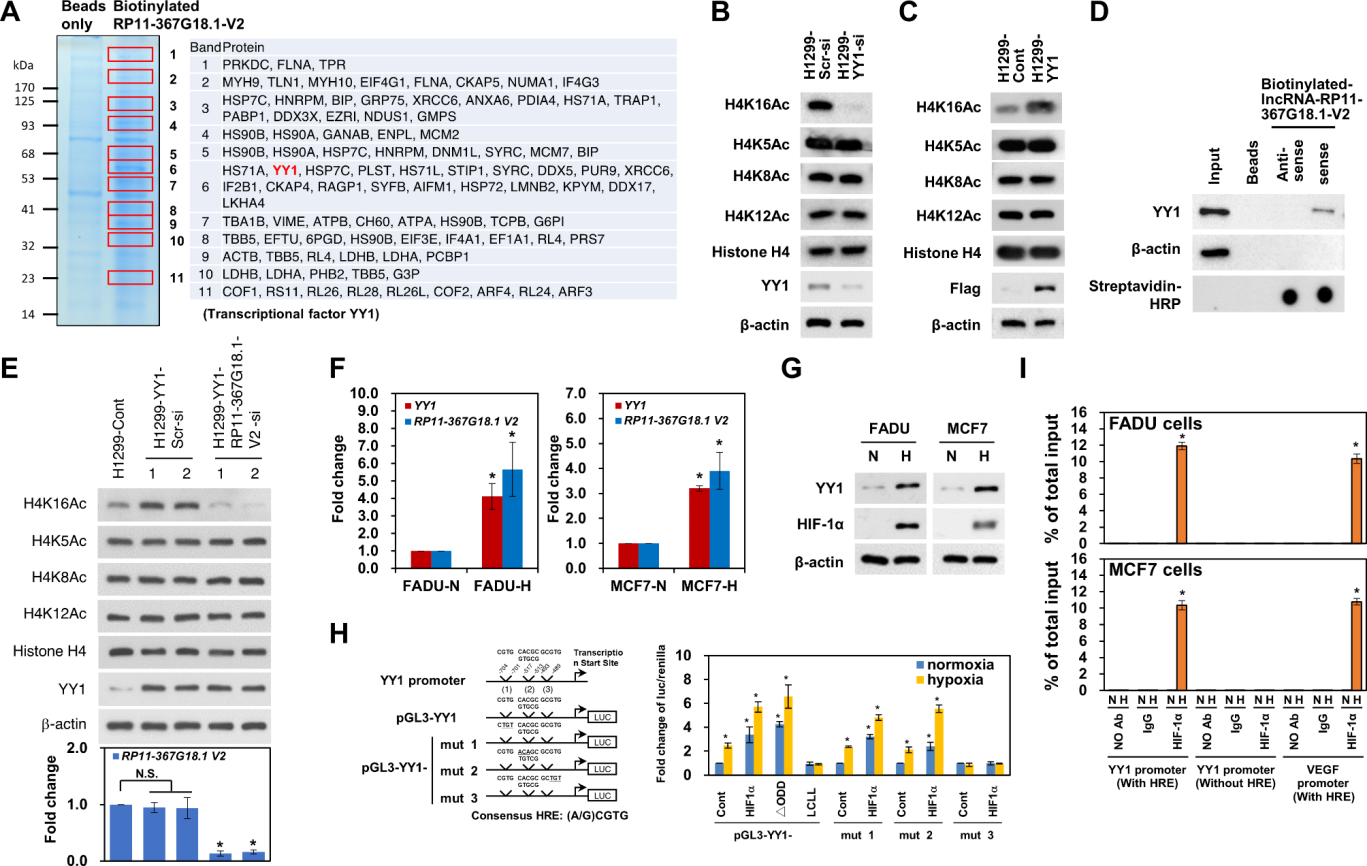
*RP11-367G18.1* variant 2 interacts with YY1 to activate H4K16Ac. (**A**) RNA pull-down assay and Coomassie blue staining revealed 11 *RP11-367G18.1* variant 2-specific bands (left). Protein identity of the 11 bands was analyzed via LC-MS/MS and was shown in a table (right). (**B**) Knockdown of YY1 suppressed H4K16Ac levels in H1299 cells. (**C**) Overexpression of YY1 increased H4K16Ac levels in H1299 cells. (**D**) YY1 was pulled down by biotinylated sense *RP11-367G18.1* variant 2. Beads or biotinylated anti-sense *RP11-367G18.1* variant 2 were used as the negative control. (**E**) YY1-induced H4K16Ac activation was suppressed following *RP11-367G18.1* variant 2 knockdown. (**F**) mRNA levels of YY1 were upregulated under hypoxia in FADU and MCF7 cells. (**G**) Protein levels of YY1 were upregulated under hypoxia in FADU and MCF7 cells. (**H**) Reporter constructs containing wild-type and mutant HREs in YY1 promoter were shown (left). Reporter assay revealed that HRE (-493/-489) was responsive to hypoxia in YY1 promoter (right). (**I**) ChIP assay revealed that HIF-1α bound to the YY1 proximal promoters containing HRE under hypoxia. For hypoxic conditions, cells were cultured in 1% O_2_, 5% CO_2_, and 94% N_2_ for 18 h. Scr, Scrambled; Cont, control; V2, variant 2. Data are represented as the mean ± SD. *P < 0.05

**Fig. 3:**
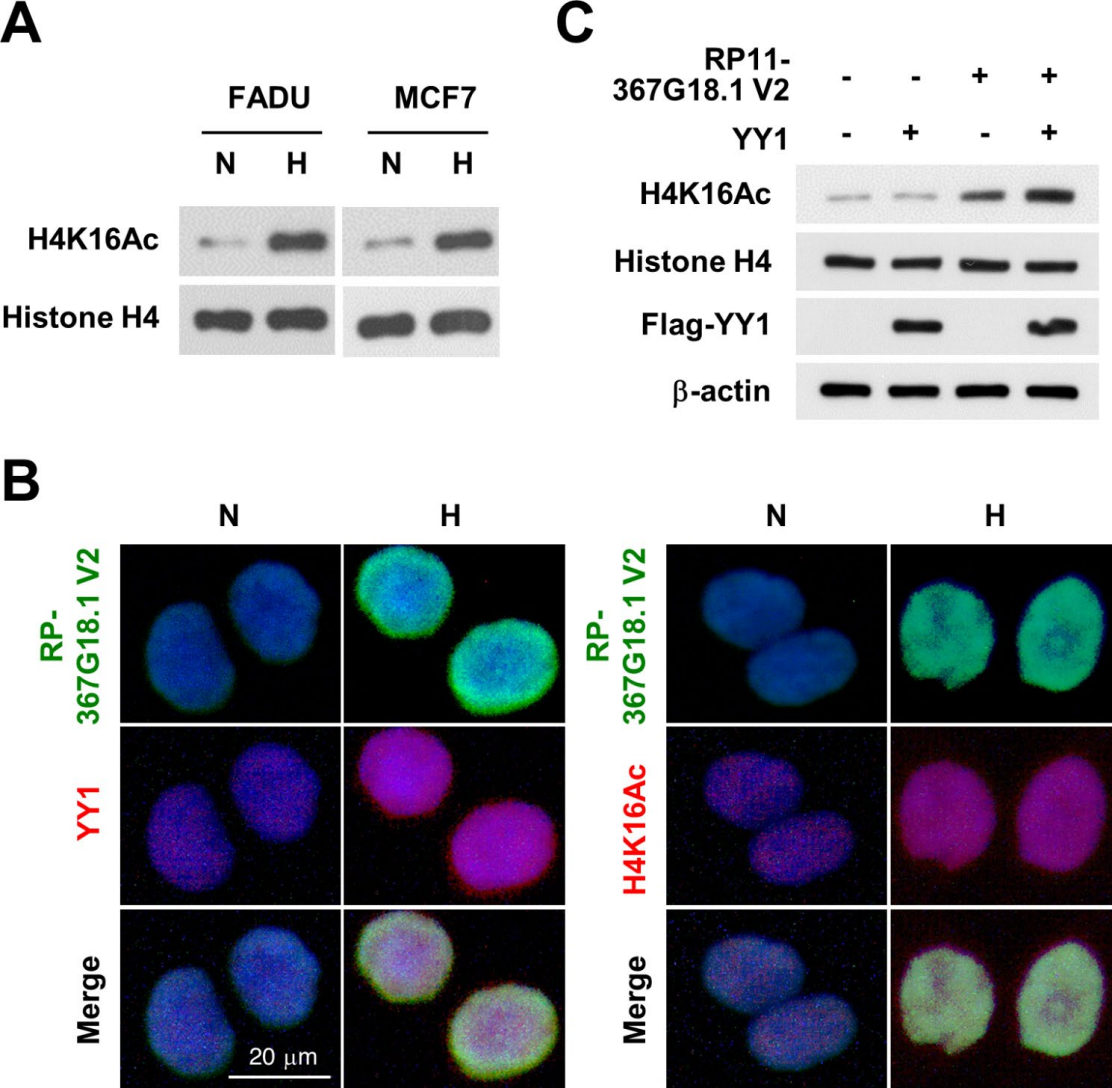
*RP11-367G18.1* variant 2-YY1 complex activates H4K16Ac. (**A**) Hypoxia enhanced H4K16Ac activation. (**B**) Immunofluorescence staining revealed that *RP11-367G18.1* variant 2 was colocalized with YY1 and H4K16Ac in FADU cells under hypoxia. (**C**) Overexpression of *RP11-367G18.1* variant 2 and YY1 enhanced H4K16Ac activation. For hypoxic conditions, cells were cultured in 1% O_2_, 5% CO_2_, and 94% N_2_ for 18 h

We previously demonstrated that *RP11-367G18.1* variant 2 specifically regulated the H4K16Ac mark [11]. To identify the binding partner of *RP11-367G18.1* variant 2 that contributed to H4K16Ac activation, we performed an RNA pull-down assay using biotinylated *RP11-367G18.1* variant 2. Biotinylated *RP11-367G18.1* variant 2 or beads (as the negative control) were incubated with whole-cell extracts of H1299 cells and pulled down using streptavidin beads. *RP11-367G18.1* variant 2-interacting proteins were analyzed using gradient gel electrophoresis and Coomassie blue staining (Fig. 2A, left). Specific bands (1–11) in *RP11-367G18.1* variant 2 pull-down samples were excised for LC-MS/MS analysis (Fig. 2A, right). YY1 has been reported to interact with histone acetyltransferases to activate gene transcription [21]. Therefore, we focused on YY1 in this study and found that the levels of H4K16Ac were decreased by YY1 knockdown and increased by YY1 overexpression in H1299 cells (Fig. 2B and C). LC-MS/MS analysis results were validated using an RNA pull-down assay. YY1, but not histone H4 or histone H3, was pulled down by biotinylated *RP11-367G18.1* variant 2. However, anti-sense RNA, *RP11-367G18.1* variant 1, or the bead control did not show any interaction with YY1 (Fig. 2D and S2A). YY1-induced H4K16Ac activation was attenuated by *RP11-367G18.1* variant 2 knockdown (Fig. 2E). *RP11-367G18.1* variant 1 and 2 were induced by hypoxia (Figure S2B). Interestingly, both mRNA and protein levels of YY1 were upregulated under hypoxic conditions (Fig. 2F and G). However, knockdown of HIF-2α did not affect the expression levels of YY1 under hypoxic conditions, indicating that HIF-1α plays a crucial role in the regulation of YY1 (Figure S2C). HIF can bind to HRE, which contains the sequence 5’-(A/G)CGTG-3’, to activate the transcription of hypoxic target genes. Three putative HREs are located in the proximal promoter of YY1. To study whether YY1 was regulated by hypoxia at the transcriptional level, reporter constructs containing the wild-type and mutant HREs in the *YY1* promoter were cloned for luciferase reporter assay (Fig. 2H, left). Reporter constructs containing wild-type HREs responded to hypoxia, HIF-1α overexpression, and constitutively active HIF-1α (ΔODD) overexpression. Reporter construct containing mutant HREs (-493 to -489 bp upstream of the transcription start site of *YY1* gene; mut3) did not respond to hypoxia and HIF-1α overexpression (Fig. 2H). HIF-1α, but not HF1-2α, bound to HREs in the *YY1* promoter under hypoxia (Fig. 2I and S2D), suggesting that *YY1* was directly modulated by HIF-1α. Moreover, cells with high endogenous HIF-1α levels exhibited high expression levels of YY1 and *RP11-367G18.1* variant 2 (Figure S1A). We observed that H4K16Ac marks were elicited by hypoxic condition (Fig. 3A). Notably, immunofluorescence staining revealed that *RP11-367G18.1* variant 2 was co-localized with YY1 and H4K16Ac in the nucleus (Fig. 3B). Knockdown of *RP11-367G18.1* variant 2 or YY1 did not have any impact on the expression levels or subcellular distributions of each other (Figure S3A and B). H4K16Ac was activated following *RP11-367G18.1* variant 2 and YY1 overexpression in FADU cells (Fig. 3C). These results indicated that the *RP11-367G18.1* variant 2–YY1 complex activated H4K16Ac under hypoxia.

### High expression levels of *RP11-367G18.1* and *YY1* are linked to worse outcomes in patients with HNSC

**Fig. 4:**
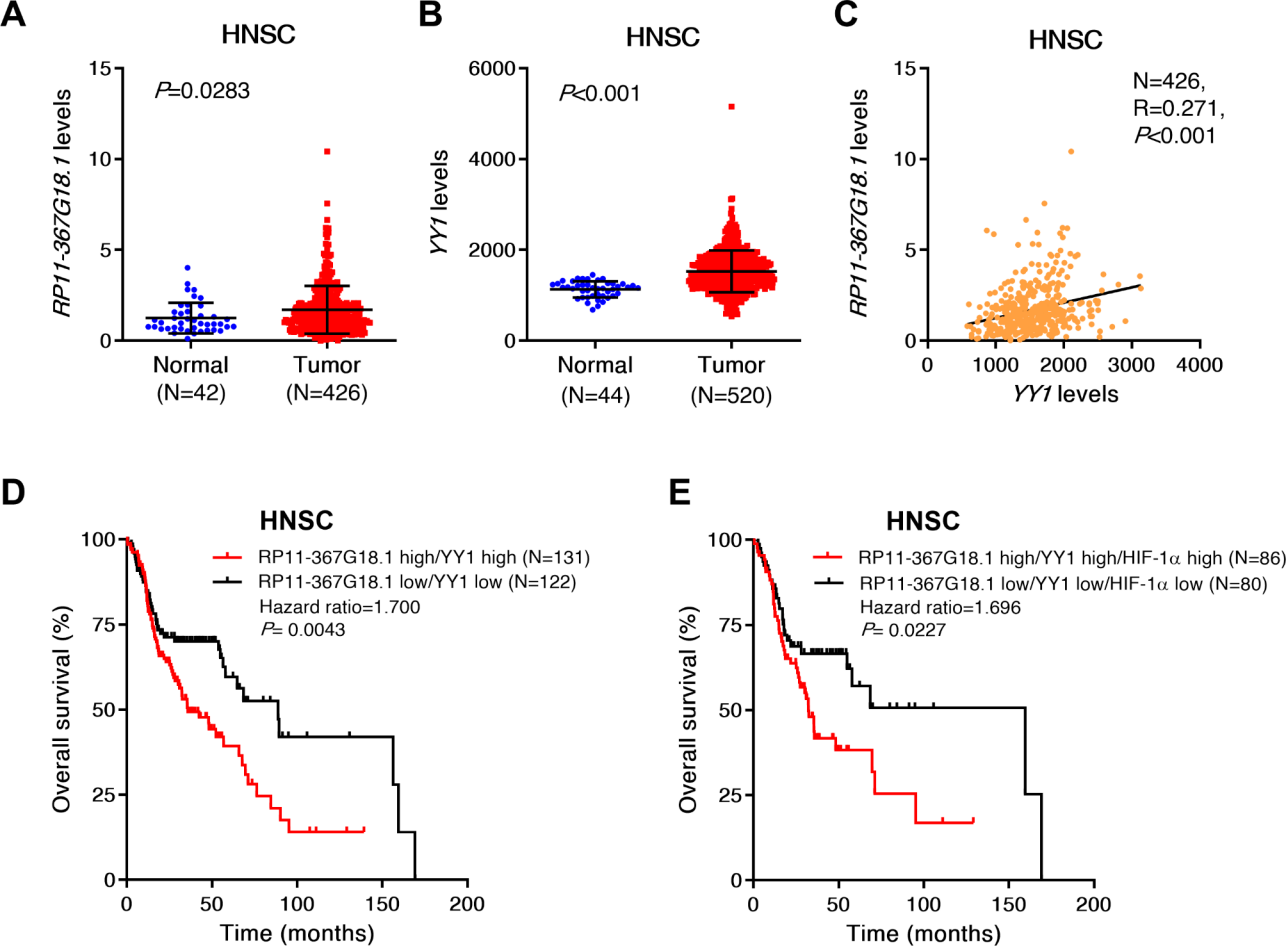
*RP11-367G18.1* and YY1 are associated with worse outcomes in patients with HNSC. (**A** and **B**) Expression levels of *RP11-367G18.1* and *YY1* were higher in tumor tissues than those in normal tissues in patients with HNSC from TCGA dataset. (**C**) Positive correlation between *RP11-367G18.1* and *YY1* in HNSC tissues was shown. (**D**) Patients with HNSC with high expression levels of *RP11-367G18.1* and *YY1* showed the worse overall survival. (**E**) Patients with HNSC with high expression levels of *RP11-367G18.1*, *YY1*, and *HIF-1α* showed the worse overall survival

We previously reported that *RP11-367G18.1* expression was associated with short survival period in patients with HNSC [11]. To further evaluate the clinical significance of *RP11-367G18.1* and *YY1*, we examined the data on gene expression and survival status using TCGA database. We found that patients with HNSC with high *YY1* expression had poor overall survival (Figure S4A). HNSC tumor tissues showed higher expression levels of *RP11-367G18.1* and *YY1* than the normal tissues (Fig. 4A and B). *YY1* expression was positively correlated with *RP11-367G18.1* and *HIF-1α* expression levels (Fig. 4C and S4B). Patients with high expression levels of both *RP11-367G18.1* and *YY1* had worse overall survival than patients with low expression levels of *RP11-367G18.1* and *YY1* (Fig. 4D and S4C). Moreover, the combination of *RP11-367G18.1*, *YY1*, and *HIF-1α* also resulted in the worse overall survival of patients with HNSC (Fig. 4E and S4D). These results highlighted the unfavorable prognostic roles of *RP11-367G18.1* and *YY1* in patients with HNSC.

### *RP11-367G18.1* variant 2–YY1 complex mediates hypoxia-induced EMT

**Fig. 5:**
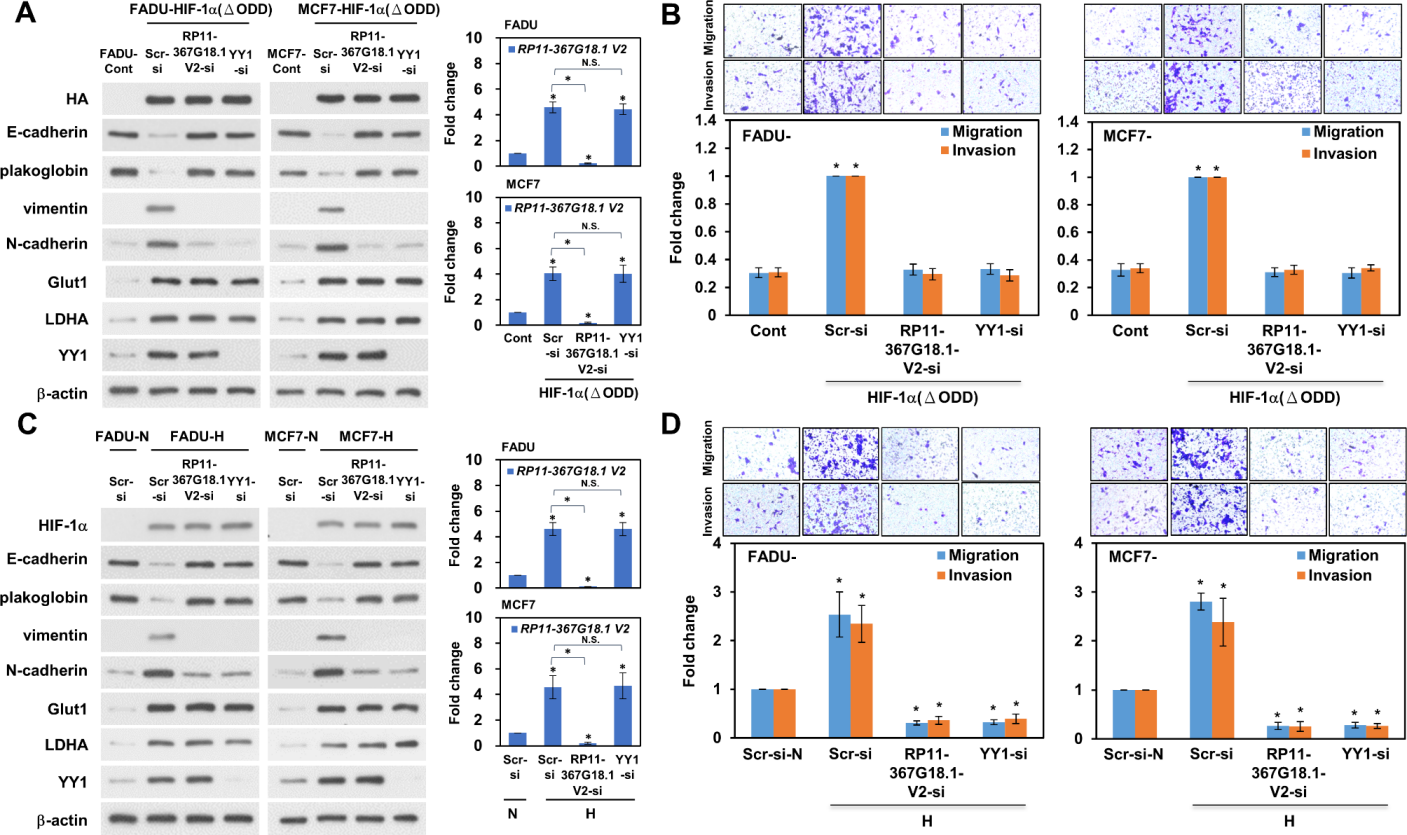
*RP11-367G18.1* variant 2–YY1 complex mediates hypoxia-induced EMT. (**A** and **B**) Knockdown of *RP11-367G18.1* variant 2 or YY1 suppressed EMT, cell migration, and invasion of FADU and MCF7 cells overexpressing HIF-1α (ΔODD). (**C** and **D**) Knockdown of *RP11-367G18.1* variant 2 or YY1 suppressed EMT, cell migration, and invasion of FADU and MCF7 cells under hypoxia. Glut1 and LDHA, the hypoxia-inducible genes, served as positive control. For hypoxic conditions, cells were cultured in 1% O_2_, 5% CO_2_, and 94% N_2_ for 18 h. Scr, Scrambled; Cont, control; V2, variant 2; N, normoxia; H, hypoxia. Data are represented as the mean ± SD. *P < 0.05

To explore the role of the *RP11-367G18.1* variant 2–YY1 complex in EMT, transwell migration and invasion assays were performed. The data revealed that *RP11-367G18.1* variant 2-enhanced cell migration and invasion were repressed by YY1 knockdown (Figure S5A). Knockdown of *RP11-367G18.1* variant 2 inhibited YY1-induced cell migration and invasion (Figure S5B). Importantly, overexpression of HIF-1α (ΔODD) suppressed the expression of epithelial markers (E-cadherin and plakoglobin), induced the expression of mesenchymal markers (vimentin and N-cadherin), and promoted cell migration and invasion. These effects were reversed by *RP11-367G18.1* variant 2 or YY1 knockdown (Fig. 5A and B). Similarly, hypoxia-induced EMT was suppressed by *RP11-367G18.1* variant 2 or YY1 knockdown (Fig. 5C and D). These results suggested that the *RP11-367G18.1* variant 2–YY1 complex mediates hypoxia-induced EMT.

### Hypoxia induces tumorigenicity via the *RP11-367G18.1* variant 2–YY1 complex

**Fig. 6:**
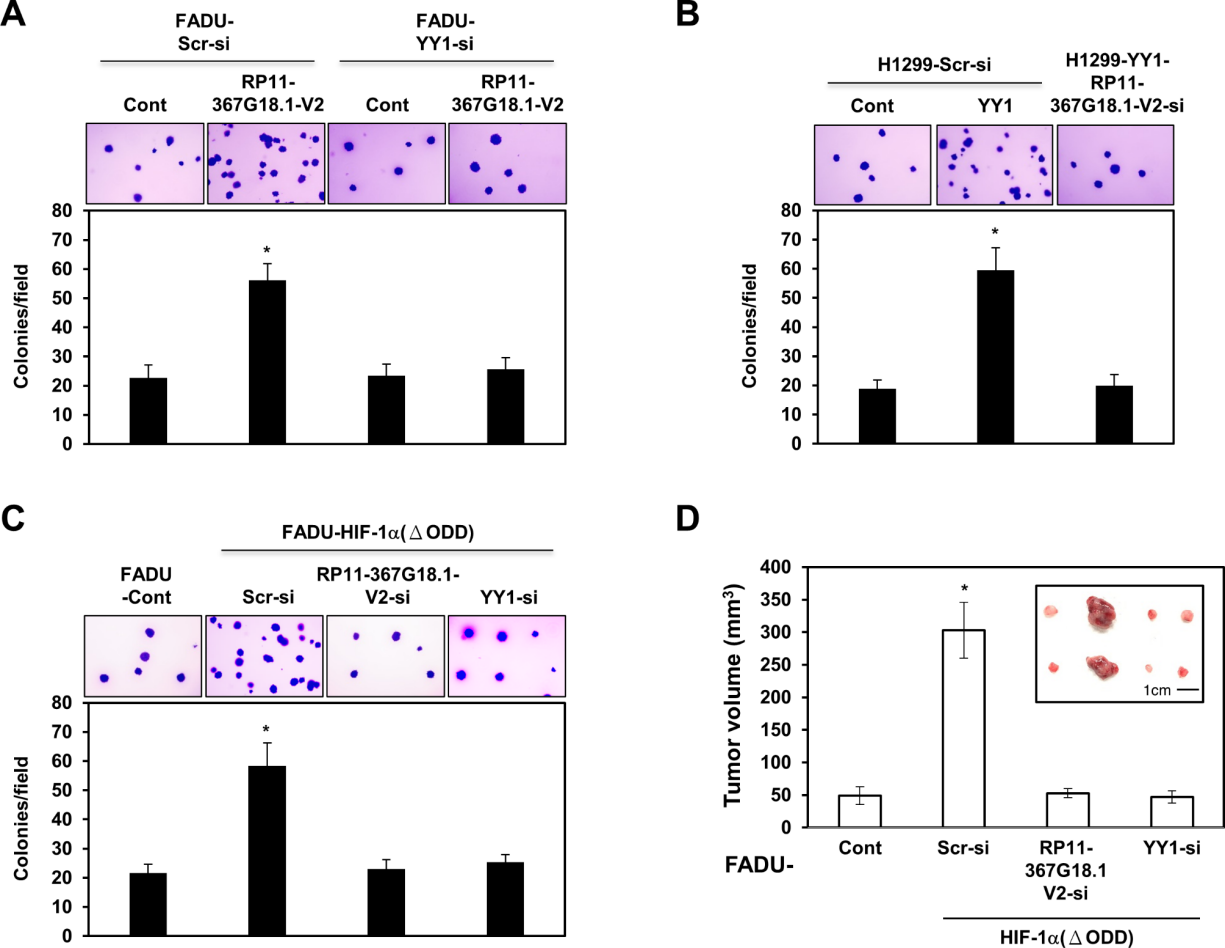
*RP11-367G18.1* variant 2–YY1 complex is essential for hypoxia-induced tumorigenicity. (**A**) Knockdown of YY1 decreased the colony formation of FADU cells overexpressing *RP11-367G18.1* variant 2. (**B**) Knockdown of *RP11-367G18.1* variant 2 suppressed the colony formation of FADU cells overexpressing YY1. (**C**) Knockdown of *RP11-367G18.1* variant 2 or YY1 suppressed the colony formation of H1299 cells overexpressing HIF-1α (ΔODD). (**D**) Knockdown of *RP11-367G18.1* variant 2 or YY1 suppressed HIF-1α (ΔODD)-enhanced tumor volume of FADU cell-derived xenografts. Scr, Scrambled; Cont, control; V2, variant 2. Data are represented as the mean ± SD. *P < 0.05

We explored the role of the *RP11-367G18.1* variant 2–YY1 complex in tumorigenicity and found that *RP11-367G18.1* variant 2-induced colony formation was inhibited by the knockdown of YY1 in FADU cells (Fig. 6A). Knockdown of *RP11-367G18.1* variant 2 inhibited YY1-enhanced colony formation in H1299 cells (Fig. 6B). Furthermore, ectopic expression of HIF-1α (ΔODD) facilitated colony formation, which was suppressed by *RP11-367G18.1* variant 2 or YY1 knockdown (Fig. 6C). We further explored the effect of the *RP11-367G18.1* variant 2–YY1 complex on hypoxia-induced tumor growth in vivo. Consistently, knockdown of *RP11-367G18.1* variant 2 or YY1 suppressed the tumor growth of xenografted FADU cells overexpressing HIF-1α (ΔODD) (Fig. 6D), suggesting that hypoxia-induced tumorigenicity was attributable to the *RP11-367G18.1* variant 2–YY1 complex.

### *RP11-367G18.1* variant 2 is essential for H4K16Ac activation and YY1 binding to the promoters of hypoxia-induced genes

**Fig. 7:**
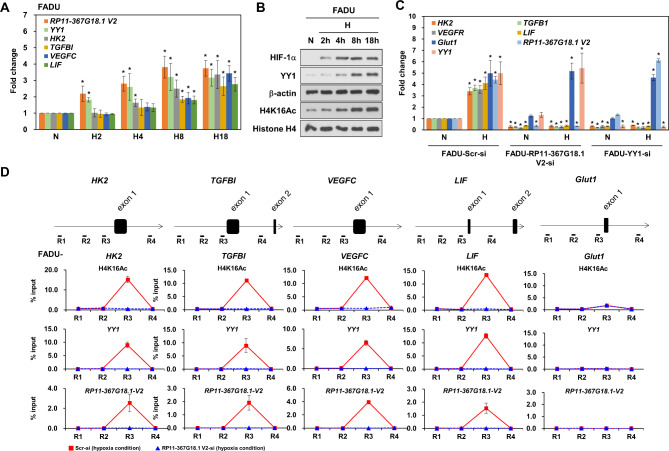
*RP11-367G18.1* variant 2–YY1 complex regulates gene expression. (**A**) The transcript expression levels of *RP11-367G18.1* variant 2, *YY1*, *HK2*, *TGFBI*, *VEGFC*, and *LIF* were measured at the indicated time points (in hours) under hypoxic conditions in FADU cells. (**B**) The proteins expressions levels of HIF-1α, YY1 and H4K16Ac were measured at the indicated time points (in hours) under hypoxic conditions in FADU cells. (**C**) Knockdown of *RP11-367G18.1* variant 2 or YY1 inhibited the expression of *HK2*, *TGFBI*. *VEGFC*, and *LIF* under hypoxia in FADU cells. (**D**) Knockdown of *RP11-367G18.1* variant 2 suppressed the level of H4K16Ac and the binding of YY1 to the proximal promoters of *HK2*, *TGFBI*, *VEGFC*, and *LIF* under hypoxic conditions. *Glut1*, a hypoxia-inducible gene, served as positive control. For hypoxic conditions, cells were cultured in 1% O_2_, 5% CO_2_, and 94% N_2_ for 18 h. Cont, control; V2, variant 2. Data are represented as the mean ± SD. Student’s *t*-test, *P < 0.05

To validate the role of the *RP11-367G18.1* variant 2-YY1 complex in regulating target genes that were co-upregulated by hypoxia and *RP11-367G18.1* variant 2 (as identified from the enriched pathways in Fig. 1E), we conducted real-time PCR analysis. Our findings revealed that the expression levels of both *RP11-367G18.1* variant 2 and YY1 transcripts were significantly elevated after a two-hour exposure to hypoxic conditions. The levels of H4K16Ac marks reached their peak after an 8-hour period of hypoxia. Subsequently, the downstream target genes were upregulated after 8 h of hypoxic exposure (Fig. 7A and B, S6A, and S6B). Hypoxia led to the upregulation of *HK2*, *TGFBI*, *VEGFC*, and *LIF*, as well as the hypoxia-inducible gene *Glut1*, which served as a positive control. Interestingly, the expression of *HK2*, *TGFBI*, *VEGFC*, and *LIF* (except for *Glut1*) was found to be suppressed upon knockdown of *RP11-367G18.1* variant 2 or YY1 (Fig. 7C and S6C). However, knockdown of *RP11-367G18.1* variant 1 did not exhibit the same effect on the expression of these genes (Figure S6D). We examined H4K16Ac levels in the promoters of *HK2*, *TGFBI*, *VEGFC*, *LIF*, and *Glut1*. ChIP assay revealed decreased levels of H4K16Ac in the promoters of *HK2*, *TGFBI*, *VEGFC*, and *LIF* under hypoxia following *RP11-367G18.1* variant 2 knockdown. Remarkably, knockdown of *RP11-367G18.1* variant 2 significantly decreased the binding of YY1 to the promoters of *HK2*, *TGFBI*, *VEGFC*, and *LIF* under hypoxia. This implies that the binding of YY1 to the promoters of hypoxia and *RP11-367G18.1* variant 2 co-upregulated genes is dependent on *RP11-367G18.1* variant 2 (Fig. 7D and S6E). The 306 hypoxia and *RP11-367G18.1* variant 2 co-upregulated genes comprised a total of 233 (76.1%) protein-coding genes (Fig. 1C and S7A). Under hypoxic conditions, 233 protein-coding genes were induced, with 136 of them (58.4%) being suppressed by YY knockdown (Figure S7B and C). These results indicated that the *RP11-367G18.1* variant 2–YY1 complex was essential for the regulation of hypoxia and *RP11-367G18.1* variant 2 co-upregulated genes.

## Discussion

Specific expression of lncRNAs under hypoxic conditions is usually linked to the clinicopathologic characteristics of solid tumors [22]. Hypoxia-responsive lncRNAs usually play oncogenic roles via diverse mechanisms. LncRNA *PDIA3P1* sponges miR-124-3p to activate NF-κB pathway to facilitate mesenchymal transition in glioma [23]. The hypoxic lncRNA, *KB-1980E6.3*, interacts with IGF2BP1 to stabilize c-Myc mRNA and maintain the stemness of breast cancer cells [24]. In addition, lncRNA variants may perform different biological functions [25]. *LncRNA-PXN-AS1* generates two lncRNA variants, *PXN-AS1-L* and *PXN-AS1-S*, via alternative splicing. *PXN-AS1-L* protects against PXN mRNA degradation, thereby promoting hepatocellular carcinoma progression. In contrast, *PXN-AS1-S* dissociates the translation elongation factors from PXN mRNA, thereby inhibiting PXN mRNA translation and suppressing tumorigenesis [26].

**Fig. 8:**
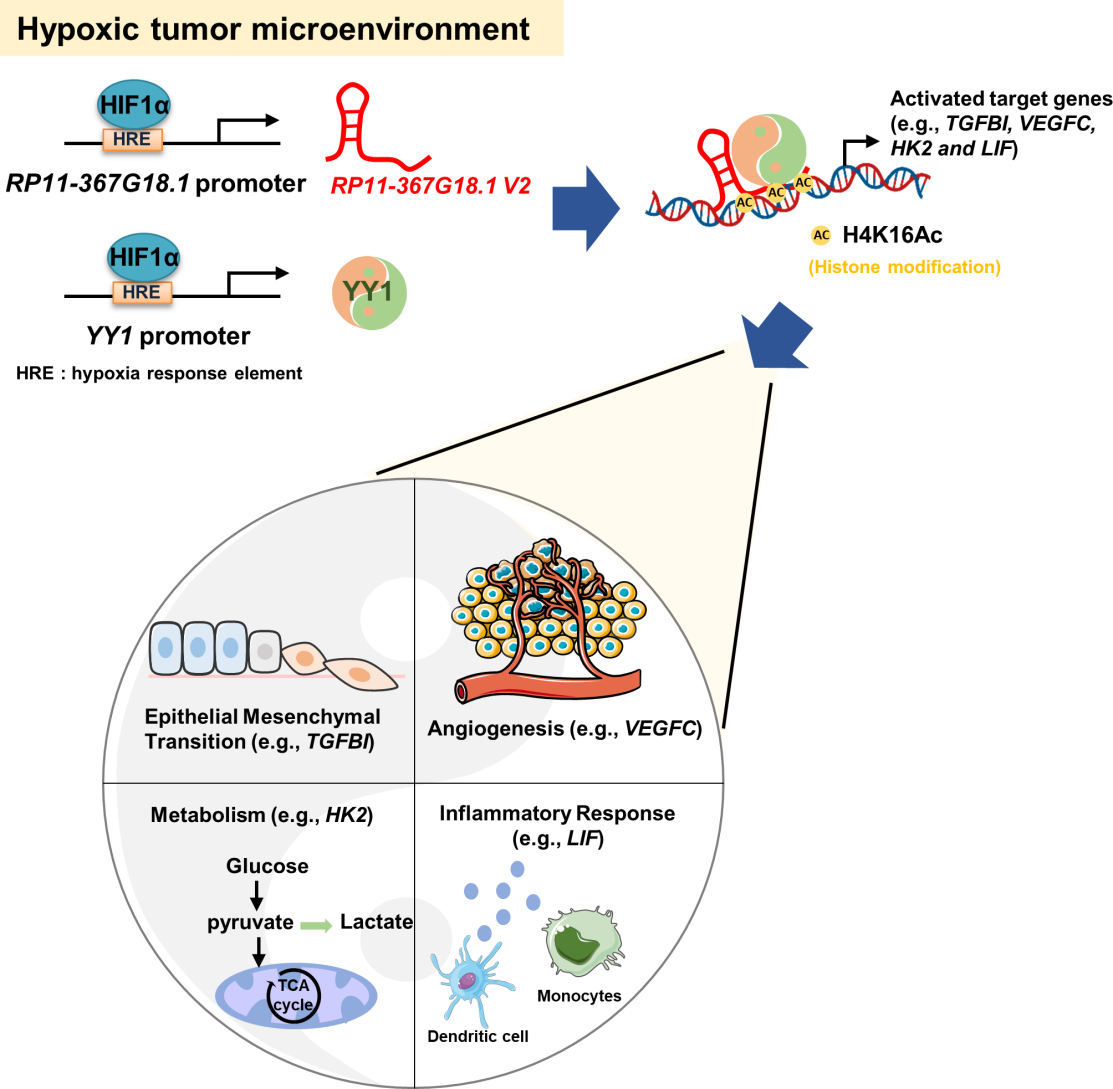
Schematic representation of the mechanism by which the *RP11-367G18.1* variant 2–YY1 complex regulates hypoxia-inducible gene expression. In a hypoxic tumor microenvironment, *RP11-367G18.1* variant 2 and YY1 are regulated by HIF-1α. *RP11-367G18.1* variant 2 and YY1 form a complex and activate H4K16Ac to increase the expression levels of hypoxia-inducible genes, *TGFBI*, *VEGFC*, *HK2*, and *LIF*

We previously reported that *RP11-367G18.1* promoted EMT and H4K16Ac activation via its variant 2, but not variant 1 [11]. In this study, we investigated the molecular mechanisms underlying the role of *RP11-367G18.1* variant 2 in epigenetic regulation. Our data suggested that YY1 interacted with the *RP11-367G18.1* variant 2. Both *RP11-367G18.1* variant 2 and YY1 were regulated by hypoxia/HIF-1α. YY1 bound to the promoter of genes and activated H4K16Ac by associating with *RP11-367G18.1* variant 2 under hypoxia. The *RP11-367G18.1* variant 2–YY1 complex regulated hypoxia-inducible genes implicated in several biological and cellular processes, such as EMT, angiogenesis, metabolism, and inflammatory responses (Fig. 8).

LncRNAs are localized in the nucleus, cytoplasm, and several cellular compartments and linked to their cellular functions. Nuclear lncRNAs may perform histone modifications or transcriptional regulation depending on their interactions with DNA, RNA, and proteins [27]. LncRNAs can recruit histone modifiers modulating gene transcription [28]. LncRNA *kcnq1ot1* interacts with histone methyltransferase G9a and the PRC2 complex to increase the trimethylation of H3K9 and H3K27, thus silencing lineage-specific transcription [29]. *LncRNA-JADE* increases the expression of Jade1, a scaffold protein of HBO1 histone acetylase, thereby inducing the acetylation of H4 [30]. Although histone H4 acetylation can be observed in the promoters of active genes, H4K16Ac is associated with both transcriptional activation and repression [31]. H4K16Ac is involved in various cellular processes, such as chromatin accessibility, DNA damage response, and autophagy [32–34]. Recent studies have suggested an association between H4K16Ac and tumorigenesis [35, 36].

Transcription factor, YY1, is ubiquitously expressed in mammalian cells and acts as a transcriptional activator and repressor [21]. Some DNA-binding transcription factors have been reported to bind to RNA [37]. YY1 binds to single-stranded RNA with low specificity [38]. YY1 can bind to both lncRNA *Xist* and DNA via different sequences, which is essential for tethering *Xist* to the inactive X nucleation center [39]. YY1 physically interacts with lncRNA *Sox2ot* to suppress the differentiation of neural progenitors by inhibiting *Sox2* expression [40]. Interestingly, both histone acetyltransferases and histone deacetylases (HDACs) interact with YY1. YY1 interacts with p300 and CREB-binding protein to activate transcription [21]. YY1 recruits HDAC2 to deacetylate histone H3, thereby suppressing chondromodulin-I expression [41]. In addition, YY1 recruits the histone H4-specific methyltransferase, PRMT1 to the YY1-activated promoter [42]. These studies suggest that YY1 regulates gene transcription in a context-dependent manner. Nevertheless, LC-MS/MS analysis revealed that no histone modifier was pulled down by biotinylated *RP11-367G18.1* variant 2. Hence, YY1-related histone modifiers mediating *RP11-367G18.1* variant 2-activated H4K16Ac need to be investigated further in future studies.

## Conclusions

In conclusion, our results revealed that the *RP11-367G18.1* variant 2–YY1 complex promotes cancer progression in a hypoxic tumor microenvironment. Moreover, the *RP11-367G18.1* variant 2–YY1 complex enhances hypoxia-inducible gene expression via H4K16Ac activation. Therefore, the *RP11-367G18.1*–YY1 complex can potentially be used as a therapeutic target for HNSC treatment.

### Electronic supplementary material

Below is the link to the electronic supplementary material.

Supplementary Material 1

Supplementary Material 2

## Data Availability

All data generated or analysed during this study are included in this published article and its supplementary information files.
