# Supplementary material for: Interplay between lncRNA RP11-367G18.1 variant 2 and YY1 plays a vital role in hypoxia-mediated gene expression and tumorigenesis
Source: Cancer Cell Int. 2023 Nov 8;23:266. doi: 10.1186/s12935-023-03067-6 (PMC10634066; doi:10.1186/s12935-023-03067-6)
Supplement: Supplementary file 1 — Supplementary Material 1 [file 12935_2023_3067_MOESM1_ESM.docx]

**Supplemental information**

**Table S1.** Sequence of the *RP11-367G18.1* variant 2 probes for RNA FISH assay

| **Probe Name** | **Sequence (5’→3’)** |
| --- | --- |
| RP11-367G18.1 V2-FISH1 | AACAGAAAGCTGATCGAGCC |
| RP11-367G18.1 V2-FISH2 | CATGGCATCGTAAGGCAATG |
| RP11-367G18.1 V2-FISH3 | CTGATTTGACAATAGTTCCA |
| RP11-367G18.1 V2-FISH4 | ACTGAAAAGCTGGGGAGTGA |
| RP11-367G18.1 V2-FISH5 | CCTCTGATAGAGCAAGAATT |
| RP11-367G18.1 V2-FISH6 | ATCATTATACTGTTGTCCTC |
| RP11-367G18.1 V2-FISH7 | TGCCAGGAAGTAGAACCTAT |
| RP11-367G18.1 V2-FISH8 | GGCCCGTGGGAAAATGATTA |
| RP11-367G18.1 V2-FISH9 | AGGTAGAGACAGTTATTTCA |
| RP11-367G18.1 V2-FISH10 | CTGTCTTTCATTCTTTCCAT |
| RP11-367G18.1 V2-FISH11 | TTCTTGCAGAGAGTTGCTTA |
| RP11-367G18.1 V2-FISH12 | AGCAGAACCTAAGCCATATG |
| RP11-367G18.1 V2-FISH13 | TGCATGGAATCAAATCACCA |
| RP11-367G18.1 V2-FISH14 | TGTCAAGTCCCGGTGCTG |
| RP11-367G18.1 V2-FISH15 | CCCATCTCAGTAGTGAATAC |
| RP11-367G18.1 V2-FISH16 | GTCTTCATATTCATCAATTC |
| RP11-367G18.1 V2-FISH17 | GTTCTATTTCTGCTTTCTTA |
| RP11-367G18.1 V2-FISH18 | TAGACAATTCGAGAAAGGTA |
| RP11-367G18.1 V2-FISH19 | CCACATAGCAAGATCCCATC |
| RP11-367G18.1 V2-FISH20 | AGGAGTTTGAGGCCAGCCTG |

**Table S2.** Sequence of the oligonucleotides for real-time PCR assay

| Target | Sequence(5’→3’) |
| --- | --- |
| *RP11-367G18.1* variant 2 | F: TACGGCTCGATCAGCTTTCTGT |
|  | R: GAAAGACTGAAAAGCTGGGGAG |
| *RP11-367G18.1* variant 1 | F: AACTCTCTGCAAGAAGACATATGGC |
|  | R: CAGATTGTACATTGAAGAGGACCTGT |
| *YY1* | F: AAGAGCGGCAAGAAGAGTTAC |
|  | R: CAACCACTGTCTCATGGTCAATA |
| *HK2* | F: GAGCCACCACTCACCCTACT |
|  | R: CCAGGCATTCGGCAATGTG |
| *TGFBI* | F: GGCCAGATCCTGTCCAAGC |
|  | R: GTGGGTTTCCACCATTAGCAC |
| *VEGFC* | F: ATGTGTGTCCGTCTACAGATGT |
|  | R: GGAAGTGTGATTGGCAAAACTGA |
| *LIF* | F: CCAACGTGACGGACTTCCC |
|  | R: TACACGACTATGCGGTACAGC |
| *Glut1* | F: CGGGCCAAGAGTGTGCTAAA |
|  | R: TGACGATACCGGAGCCAATG |
| *18S* | F: GGCGGCGTTATTCCCATGA |
|  | R: GAGGTTTCCCGTGTTGAG |

**Table S3.** Sequence of the *RP11-367G18.1* variant 2 and *lacZ* probes for ChIRP assay

| **Probe Name** | **Sequence (5’→3’)** |
| --- | --- |
| RP11-367G18.1 V2-ChIRP1 | TCATGGCATCGTAAGGCAAT |
| RP11-367G18.1 V2-ChIRP2 | CATTATACTGTTGTCCTCGA |
| RP11-367G18.1 V2-ChIRP3 | AGGTAGAGACAGTTATTTCA |
| RP11-367G18.1 V2-ChIRP4 | CACCAATAGCTCAGCAGAAC |
| RP11-367G18.1 V2-ChIRP5 | CGTAGGGCTGTCAAGTCC |
| RP11-367G18.1 V2-ChIRP6 | GCTGGGGAGTGAACTCTG |
| RP11-367G18.1 V2-ChIRP7 | GGCCCGTGGGAAAATGAT |
| RP11-367G18.1 V2-ChIRP8 | GCCATATGTCTTCTTGCA |
| lacZ-ChIRP1 | TAAATGTGAGCGAGTAACAACC |
| lacZ-ChIRP2 | TGCCATAAAGAAACTGTTACCC |
| lacZ-ChIRP3 | GAAGGATCGACAGATTTGATCC |
| lacZ-ChIRP4 | ATTTAATCAGCGACTGATCCAC |
| lacZ-ChIRP5 | GTCAGCAGTTGTTTTTTATCGC |

**Table S4.** Sequence of the oligonucleotides for qChIP and qChIRP assays

| Target | Sequence(5’→3’) |
| --- | --- |
| *YY1* (with HRE) | F: GGTTTTGTGGCTGTTGCACC |
|  | R: AATCGATCTGTCCGCTGGC |
| *YY1* (without HRE) | F: AGTTCTCCCACAACTGGTTTCC |
|  | R: TTGCTAAATCGCATTGGACTTG |
| *VEGF* (with HRE) | F: ACAGACGTTCCTTAGTGCTGG |
|  | R: AGCTGAGAACGGGAAGCTGTG |
| *HK2* (R1) | F: ATAAGCCTGCAACTCCAGGAGA |
|  | R: CCTCCACCCTCTTCTCTGAACTAG |
| *HK2* (R2) | F: AAGCTATCCTTCCACCTTAGCCTT |
|  | R: GTTGCTTGAGACCAGGAGTTCAT |
| *HK2* (R3) | F: AGAGGCCCGTTTTTCCAGTC |
|  | R: CCTGAGATGGGACGTGTGGT |
| *HK2* (R4) | F: TCCAAATCAGCCTCGGGAC |
|  | R: GAGCCAGGTGGGAACAGACA |
| *TGFBI* (R1) | F: GCCATCCATGTTCATGTCTGC |
|  | R: TTCTTGCCCCTCTCTAATCCAC |
| *TGFBI* (R2) | F: AATCAGCAGGAGTCTGGTCCC |
|  | R: GCTACTGGGCACATGGCAA |
| *TGFBI* (R3) | F: AGATGAGGACGGTGGCCC |
|  | R: GGTCGGCAGGGGGTTTT |
| *TGFBI* (R4) | F: AAGTGACTGGCAGAAGAAACTGG |
|  | R: CGCATGTTTCTGTCGCACTC |
| *VEGFC* (R1) | F: ATGCTTTTCAGCAGTGTTGCTC |
|  | R: CTCACCCATAATTTACTTGAAGGCT |
| *VEGFC* (R2) | F: ACCAACTTCTTGCAGAAGCTGAC |
|  | R: CTGTTGGGATGGATGCTAATGA |
| *VEGFC* (R3) | F: CATCACCTCTAAAGCCGGTCC |
|  | R: GCCTGCGCTTATGTGAGAGAA |
| *VEGFC* (R4) | F: AGAACCCGAGAGTTTGCGTTAG |
|  | R: TCGTGTAACTTGCTTGCCTCTC |
| *LIF* (R1) | F: AAGGATGCAGGGGAGACTGG |
|  | R: TGCCACCTGACTCAGCCTG |
| *LIF* (R2) | F: AAATCGCCTGGCCTCAGAA |
|  | R: ACTGAGAGAGGCTTGGCGAA |
| *LIF* (R3) | F: ATCTTCAGACAACTCCCGGGA |
|  | R: AGTCCAGGAAGTTGTTTGAGGTG |
| *LIF* (R4) | F: ACCAGGACATGTCGGGACA |
|  | R: TCCCTCCTAACACACACACACC |
| *Glut1* (R1) | F: TGGGAAAAGGCATAGACTGG |
|  | R: ATGCACGAATGAGTGAGCAG |
| *Glut1* (R2) | F: CTTGAGCCCAGGAGTTTGAG |
|  | R: GGAGAGGTGCAATTTCCAGA |
| *Glut1* (R3) | F: TGGTTCAAACCCGAGGTCTA |
|  | R: GGGACGCCTTCCTCTACTTC |
| *Glut1* (R4) | F: CCTGCAGGGCATCTTTGTAG |
|  | R: GGAGGAAAGGAGGTGGAAAG |

**
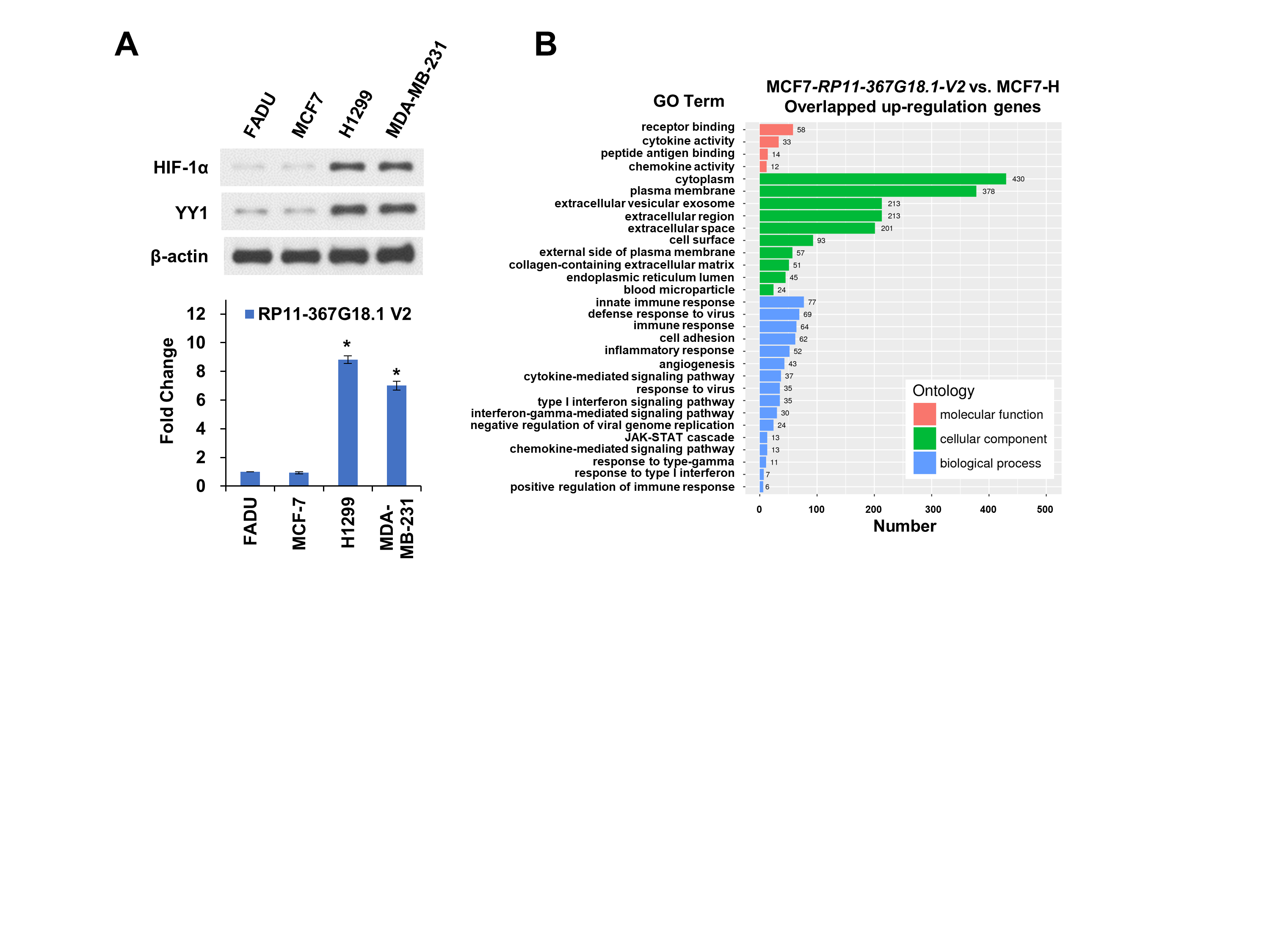
**

**Figure S1.** **The expression levels of HIF-1α, YY1, and *RP11-367G18.1* variant 2 in cancer cells as well as gene ontology analysis of *RP11-367G18.1* variant 2 and hypoxia upregulated genes.**

(A) The expression levels of HIF-1α, YY1, and *RP11-367G18.1* variant 2 in cancer cells. (B) As shown in Figure 1C, bar charts showed gene ontology analysis of hypoxia and *RP11-367G18.1* variant 2 co-upregulated 306 genes. **P* < 0.05.


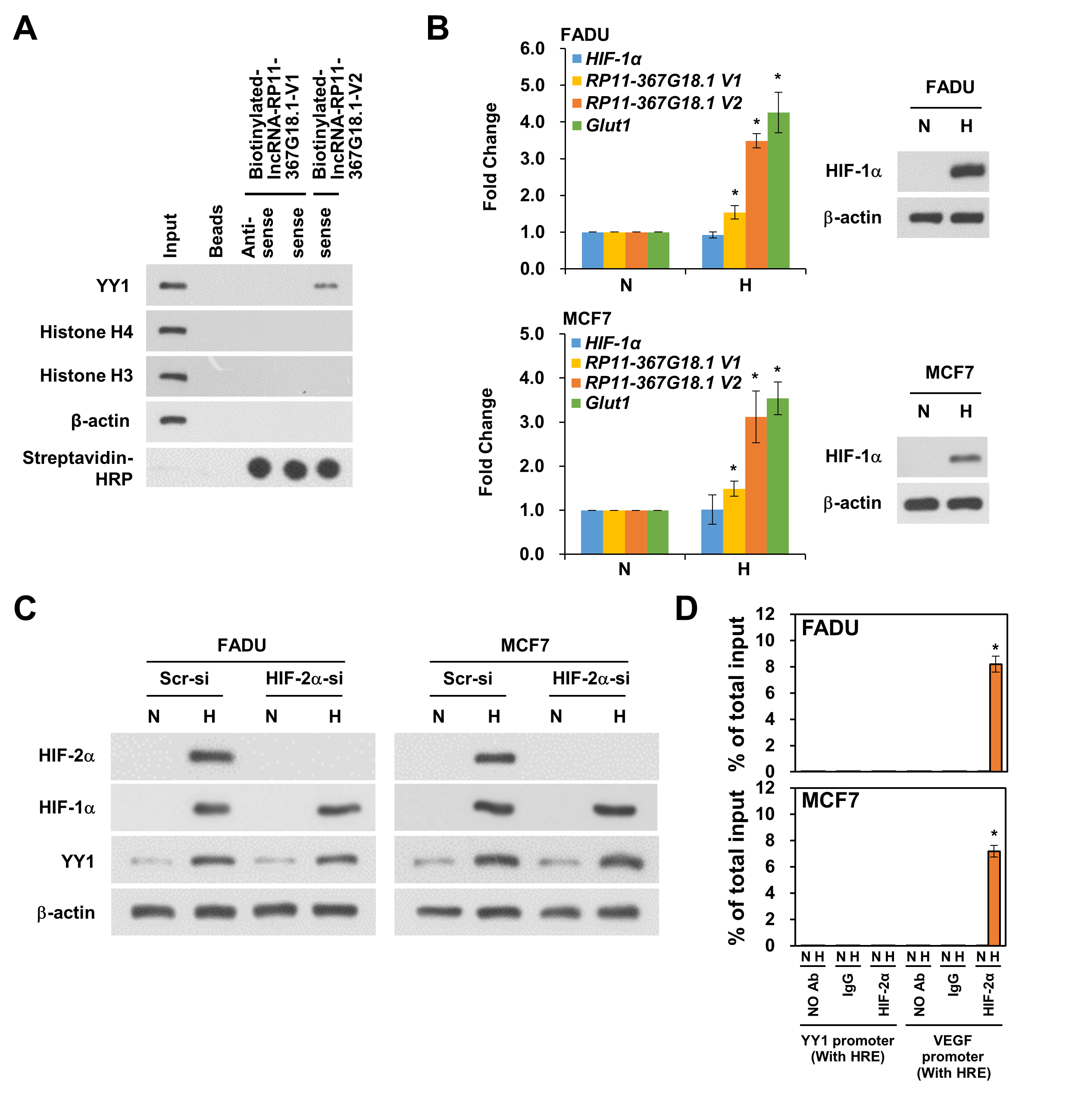


**Figure S2. Related to Figure 2.**

(A) YY1 was pulled down by biotinylated sense *RP11-367G18.1* variant 2 but not variant 1. Beads or biotinylated anti-sense *RP11-367G18.1* variant 1 were used as the negative control. (B) *RP11-367G18.1* variant 1 and 2 were upregulated under hypoxia in FADU and MCF7 cells. *Glut1*, a hypoxia-inducible gene, served as positive control. (C) Knockdown of HIF-2α did not affect hypoxia-induced YY1 expression. (D) HIF-2α did not bind to YY1 promoter. For hypoxic conditions, cells were cultured in 1% O_2_, 5% CO_2_, and 94% N_2_ for 18 h. Scr, scramble; V1, variant 1; V2, variant 2; N, normoxia; H, hypoxia. Data are represented as the mean ± SD. **P* < 0.05.


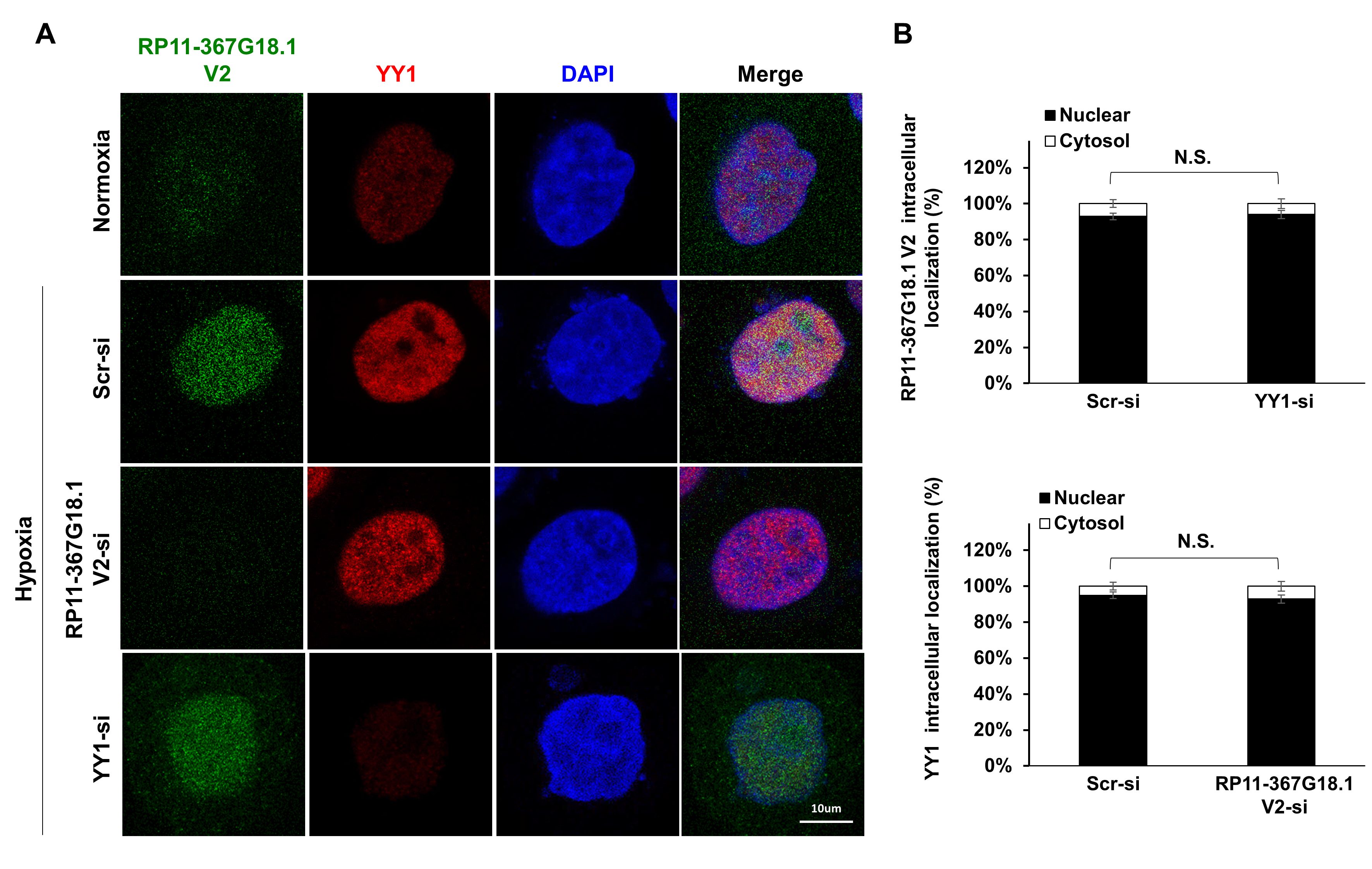


**Figure S3. Subcellular distributions of *RP11-367G18.1* variant 2 and YY1.**

(A) Immunofluorescence staining revealed that knockdown of *RP11-367G18.1* variant 2 or YY1 did not affect the expression and localization of each other. (B) Subcellular distributions of *RP11-367G18.1* variant 2 and YY1 in FADU cells under hypoxia. N.S., not significant.


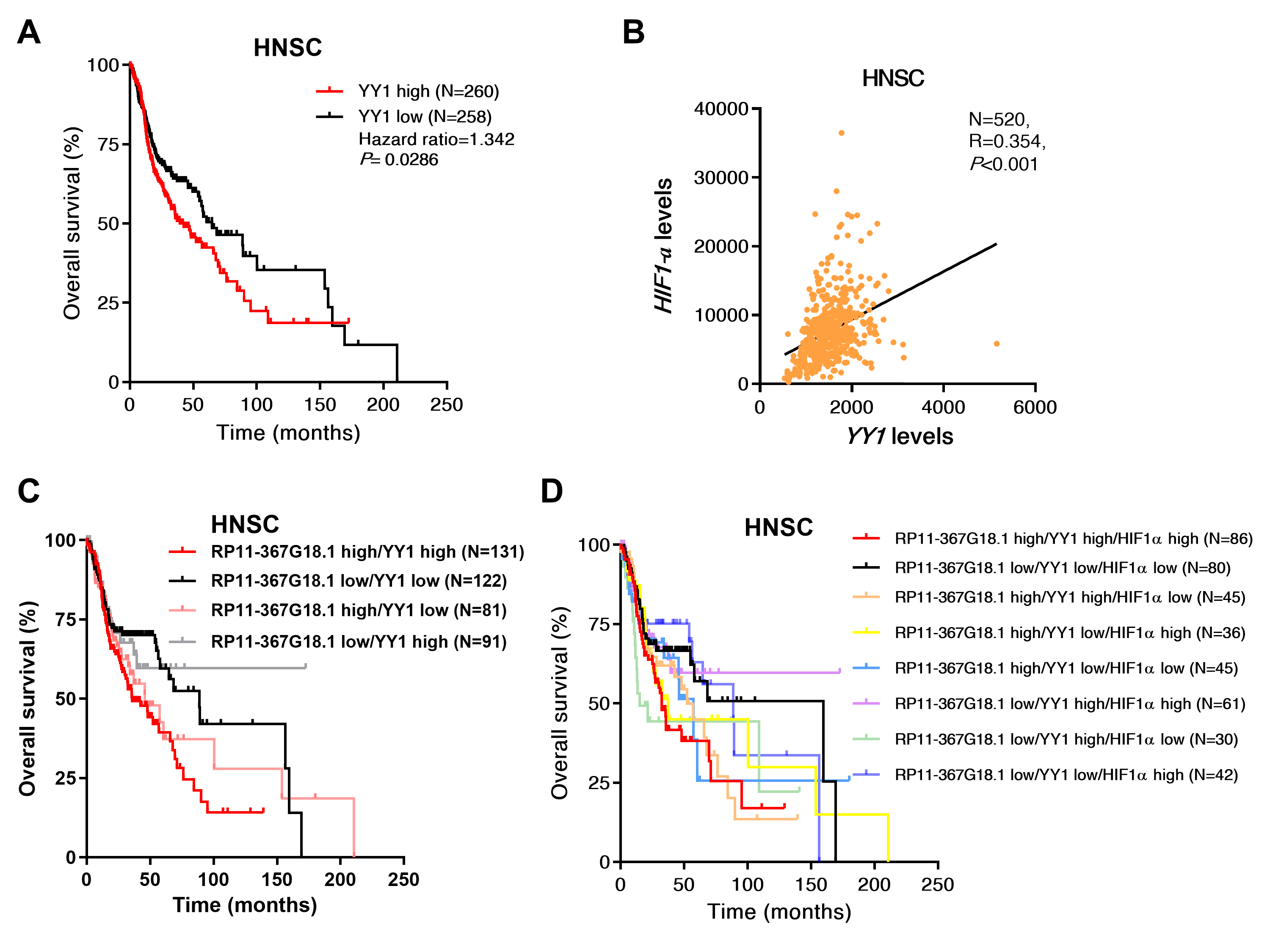


**Figure S4. YY1 expression is associated with worse survival in patients with HNSC.**

(A) HNSC patients with high *YY1* expression had inferior overall survival. (B) *YY1* expression was positively correlated with *HIF-1α* expression in HNSC tissues. (C and D) Related to Figure 4D and E. The association between gene expression and overall survival of HNSC patients. The HNSC patients were divided into subgroups based on the expression levels of *RP11-367G18.1*, *YY1*, and *HIF-1α*.


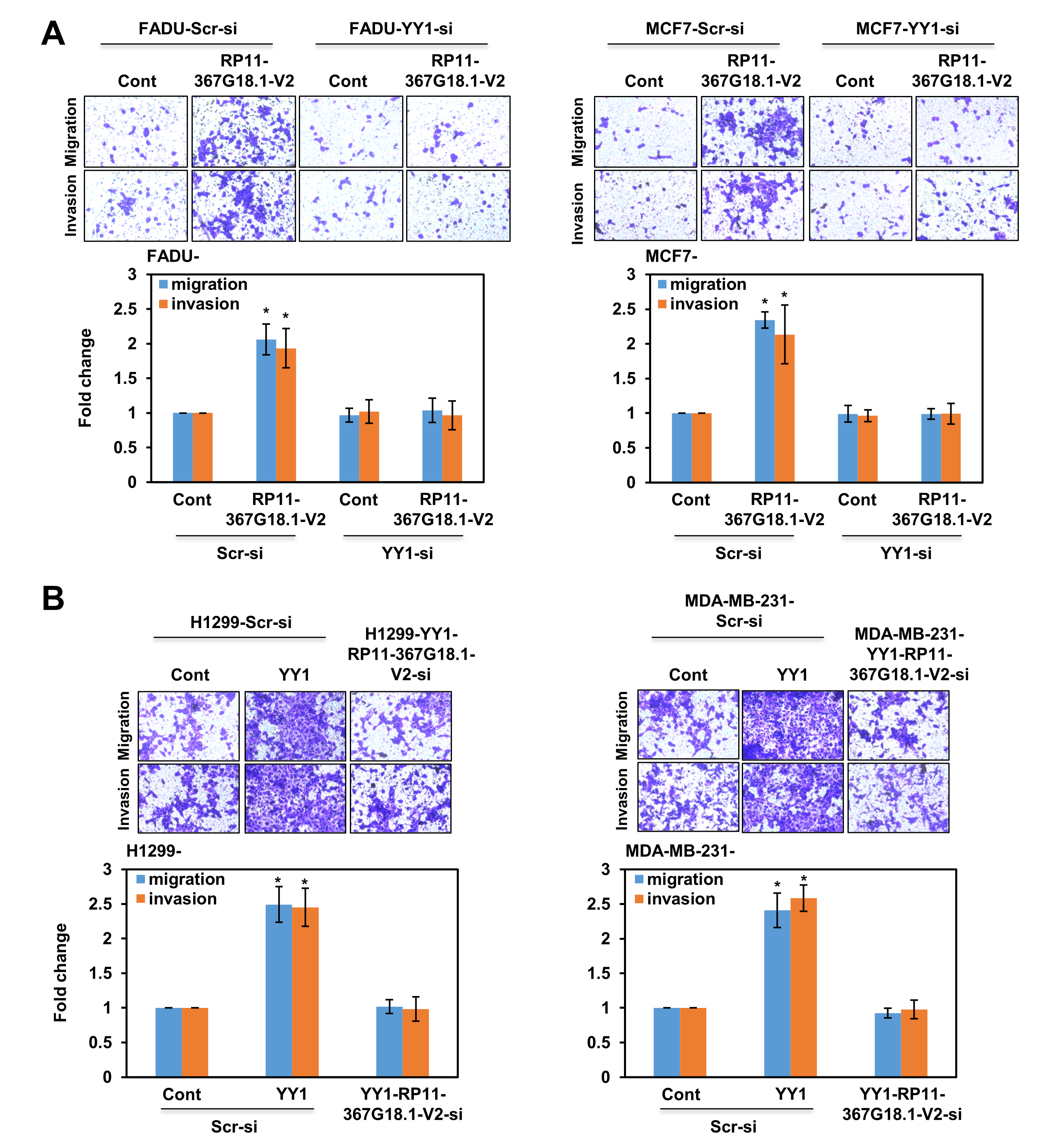


**Figure S5. *RP11-367G18.1* variant 2-YY1 complex enhances cell migration and invasion.**

(A) Knockdown of YY1 suppressed cell migration and invasion of FADU and MCF7 cells that overexpressing *RP11-367G18.1* variant 2. (B) Knockdown of *RP11-367G18.1* variant 2 suppressed cell migration and invasion of H1299 and MDA-MB-231 cells that overexpressing YY1. Scr, scramble; Cont, control; V2, variant 2. Data are represented as mean ± SD. *, *P* < 0.05.


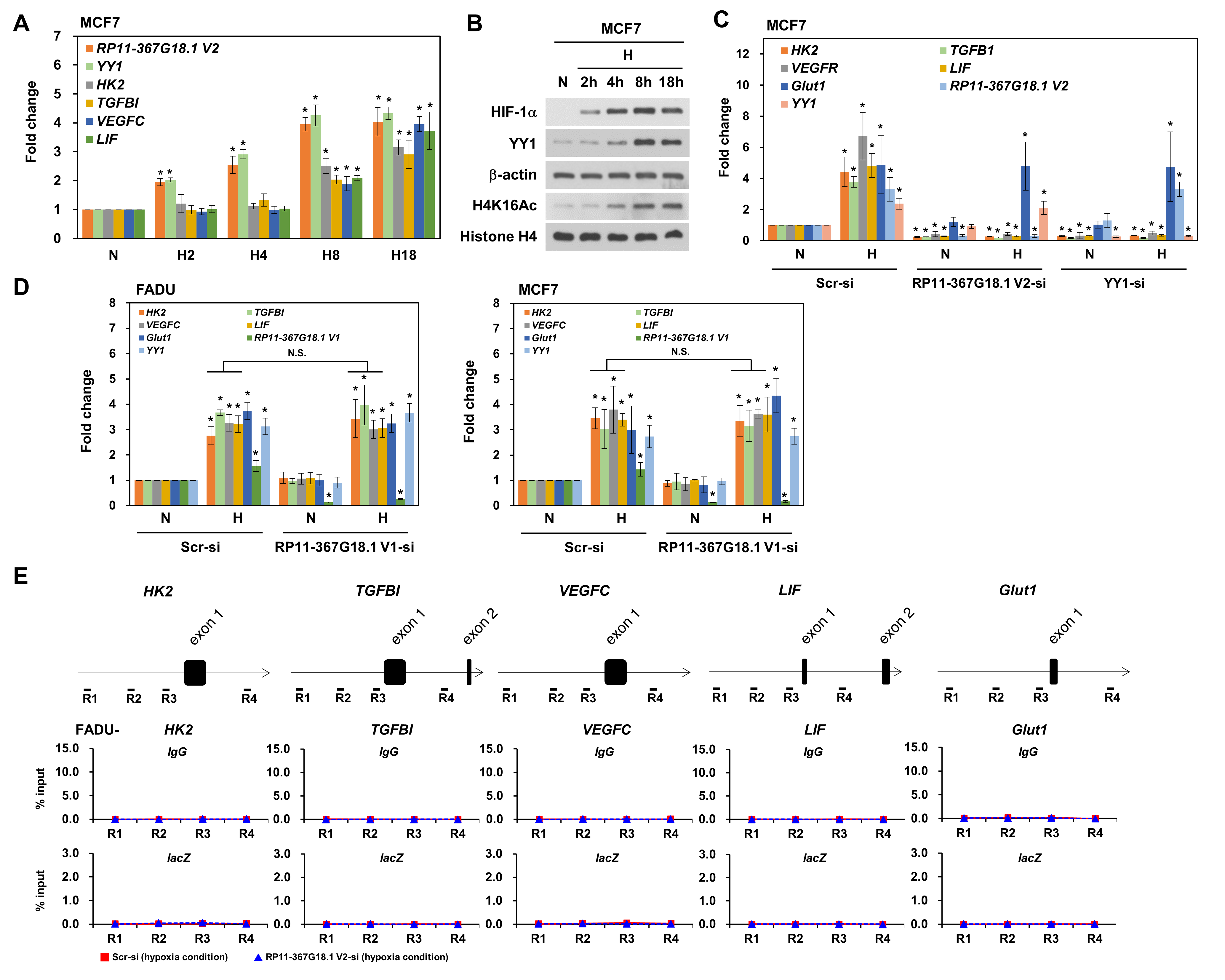


**Figure S6. Related to Figure 7.**

(A) The transcript expression levels of *RP11-367G18.1* variant 2, *YY1*, *HK2*, *TGFBI*, *VEGFC*, and *LIF* were measured at the indicated time points (in hours) under hypoxic conditions in MCF7 cells. (B) The proteins expressions levels of HIF-1α, YY1 and H4K16Ac were measured at the indicated time points (in hours) under hypoxic conditions in MCF7 cells. (C) Knockdown of *RP11-367G18.1* variant 2 or YY1 inhibited the expression of *HK2*, *TGFBI*. *VEGFC*, and *LIF* under hypoxia in MCF7 cells. (D) Knockdown of *RP11-367G18.1* variant 1 did not affect the expression of *HK2*, *TGFBI*. *VEGFC*, *LIF*, *Glut1*, and *YY1* under hypoxia in FADU and MCF7 cells. (E) Related to Figure 7D. The IgG and *lacZ* control groups for ChIP and ChIRP assays, respectively. For hypoxic conditions, cells were cultured in 1% O_2_, 5% CO_2_, and 94% N_2_ for 18 h. N, normoxia; H, hypoxia. Data are represented as the mean ± SD. **P* < 0.05. N.S., not significant.


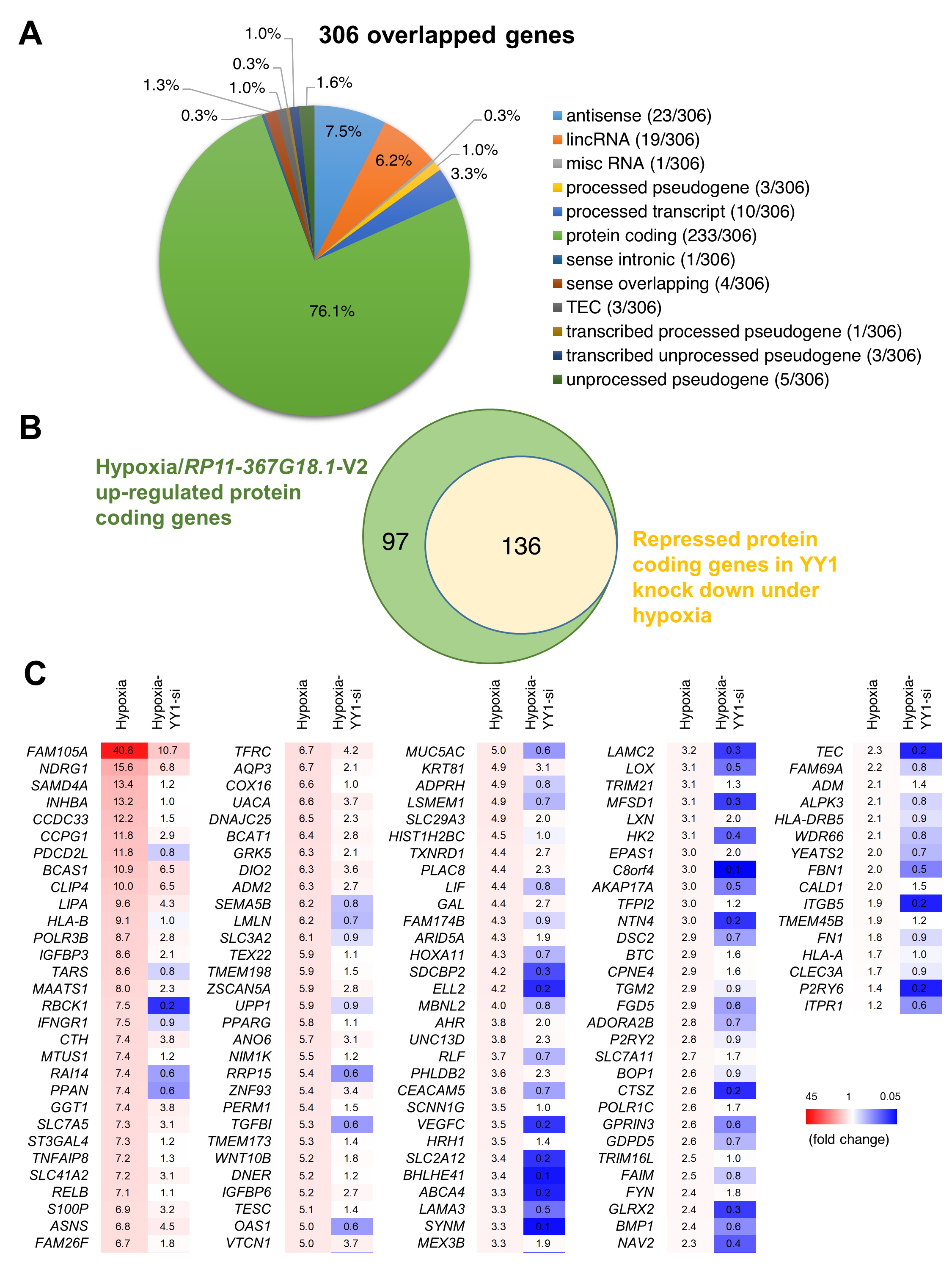


**Figure S7. YY1 regulates hypoxia and *RP11-367G18.1* variant 2 co-upregulated genes.**

(A) Related to Figure 1C. Pie chart of the percentage of the 306 hypoxia and *RP11-367G18.1* variant 2 co-upregulated genes. (B) 136 out of 223 hypoxia and *RP11-367G18.1* variant 2 co-upregulated protein-coding genes were regulated by YY1. (C) The fold change of 136 protein-coding genes in both the control and YY1 knockdown conditions under hypoxia.
